# How Do Social Network Sites Influence Workout Intentions: A Social Norm Approach

**DOI:** 10.3389/fpsyg.2021.753189

**Published:** 2021-11-23

**Authors:** Jian Raymond Rui, Shuangqing Liu

**Affiliations:** ^1^School of Journalism and Communication, South China University of Technology, Guangzhou, China; ^2^Center for Public Health Risk Surveilance and Information Communication, Guangzhou, China; ^3^Guangming School of Journalism and Communication, China University of Political Science and Law, Beijing, China

**Keywords:** SNS, perceived injunctive norm, perceived descriptive norm, workout, self-efficacy, psychological distance

## Abstract

People share their workout experiences on social network sites (SNSs). The present study examined how perceived exposure to these workout-related SNS posts may affect individuals’ engagement in physical activities through perceived descriptive and injunctive norms of workout in their network, and how self-efficacy in workout moderated the effect of perceived descriptive norm on their workout intention, which was measured in general and specific ways. An online survey was conducted with a convenience sample of 807 responses in China. Results show that perceived descriptive norm of workout in one’s network mediated the relationship between perceived exposure to workout-related SNS posts and perceived injunctive norm. In addition, self-efficacy in workout moderated the effect of perceived descriptive norm on workout intention—both general and specific—but the normative influence was stronger at a low level of self-efficacy compared to a high level. Furthermore, perceived injunctive norm only predicted the general rather than specific workout intention, suggesting that the perception of most people’s approval might not be priority when people consider details about workout. These findings develop the theory of normative social behavior by illustrating the relationship between perceived descriptive and injunctive norm and shed light on the relative strength of the motivating factors of workout in different situations.

## Introduction

The penetration rate of social network sites (SNSs) has increased rapidly across the world. By July 2020, 51% of people in the world used at least one SNS and the number of active SNS users reached 3.96 billion (Kemp, 2020). SNSs enable individuals to interact with their social contacts by sharing details of their daily life. Consequently, sharing workout experiences through SNSs has become increasingly common (Vaterlaus et al., 2015; Pinkerton et al., 2017; Liu and Kashian, 2020).

The extant scholarship has explained how workout posts *via* SNSs might motivate individuals to engage in physical activities from multiple perspectives such as social comparison (Burke and Rains, 2018; Peng et al., 2019), behavioral modeling (Vaterlaus et al., 2015; Raggatt et al., 2018), and social support (Hamari and Koivisto, 2015). The perspective of particular interest to the current study is social norm. The theory of normative social behavior (TNSB; Rimal and Lapinski, 2015) posits that individuals’ behavior is guided by their perceived social norms, which are usually categorized into descriptive versus injunctive norms (Cialdini et al., 1990). Research has extended this theory by linking perceived exposure to the information about the given behavior and perceived social norms. These studies show that the influence of SNS posts on health behavior was mediated by individuals’ perceived social norms of the given behavior, specifically perceived injunctive norm (Nesi et al., 2017; Rui and Stefanone, 2017; Yang and Zhao, 2017), perceived descriptive norm (Litt and Stock, 2011; Duong and Liu, 2019), or both (Zhang and Jemmott, 2014).

Notably, research reviewed above focuses on questionable behaviors such as drinking alcohol (Nesi et al., 2017; Rui and Stefanone, 2017; Yang and Zhao, 2017), smoking cigarettes (Litt and Stock, 2011; Duong and Liu, 2019), and sexual encounter (Zhang and Jemmott, 2014). However, the present study focuses on workout, a pro-health behavior. Although research offered empirical evidence on the facilitating effects of perceived descriptive and injunctive norms on pro-health behavior such as healthy diet (Mollen et al., 2013; Staunton et al., 2014), whether workout posts *via* SNSs can shape these two types of perceived norm, especially perceived injunctive norms, is understudied. While individuals may need SNS posts, which provide cues for approval (i.e., perceived injunctive norm), as justifications for questionable behaviors, they may not need to estimate perceived injunctive norm of pro-health behaviors based on SNS posts. This renders whether perceived exposure may still affect perceived injunctive norm of workout unknown. Following TNSB (Rimal and Lapinski, 2015), we argue that the perceived exposure to workout posts *via* SNSs may still predict perceived descriptive norm of workout directly, but its relationship with perceived injunctive norm of workout might be indirect *via* perceived descriptive norm. The first goal of this study is to test this potential mediation effect.

In addition, the main effect of self-efficacy on workout has received sufficient support (Marcus et al., 1992; Oman and King, 1998; McAuley et al., 2006; Shin et al., 2006; Haas, 2011). The present study aims to examine whether self-efficacy may moderate the relationship between perceived descriptive norm and workout intention. Rimal and Lapinski (2015) called for research examining moderators on the relationship between perceived social norm and behavior. Research on TNSB has provided empirical evidence on the moderation effect of group identity (Yang and Zhao, 2017) and outcome expectancy (Dieterich et al., 2013) for this relationship. We seek to extend this scholarship by testing the potential moderation effect of self-efficacy. Furthermore, we distinguish from previous research by using two measures of workout intention: general versus specific measures. As the construal level theory (CLT) of psychological distance shows, decisions are driven more by feasibility perceptions when individuals have a low psychological distance of the target, but desirability perceptions motivate decisions more when they exhibit a high psychological distance of the target (Liberman and Trope, 1998; Eyal et al., 2009; Chu and Yang, 2020). Thus, the relative effects of self-efficacy—as the indicator of feasibility perception—and perceived social norm—as the indicator of desirability perception—on workout intention may vary depending on the measure of intention. The literature review is structured in line with these two goals.

### Perceived Social Network Site Exposure and Workout Norm

#### Conceptualizing Social Norm

The social influence literature posits that the social norm is an important predictor of human behavior (Cialdini et al., 1990; Borsari and Carey, 2001) because norms function as codes that “either prescribe or proscribe behaviors that members of a group can enact” (Lapinski and Rimal, 2005; p. 129). As social belongingness is a fundamental need (Leary and Baumeister, 2000), social norm can pressure individuals to engage in certain behavior such as workout (Okun et al., 2002; Lee, 2011).

The theory of normative social behavior contends that different types of social norm need to be distinguished (Lapinski and Rimal, 2005). First, social norms can exist at the collective level, providing individuals in a group, community, or society with guidance on how to act in specific situations (Lapinski and Rimal, 2005). However, individuals may have different interpretations of *collective norm*, and these interpretations do not necessarily match collective norms (Borsari and Carey, 2003). Hence, norms can also exist at the individual level, which TNSB termed as *perceived norm*. The majority of research demonstrated the effect of perceived rather than actual norm on behavior (Ajzen and Fishbein, 1980; Perkins and Wechsler, 1996; Rimal and Real, 2003) because individuals tend to act based on their interpretation of the reality (Perkins and Wechsler, 1996). Thus, we focus on perceived social norm in this study.

Second, perceived norm can be categorized as perceived descriptive versus injunctive norm (Cialdini et al., 1990). While perceived descriptive norm describes one’s perception about the prevalence of the given act, perceived injunctive norm refers to the extent to which individuals think the majority approves the given act (Cialdini et al., 1990). Both norms can influence behavior, but the mechanisms of their influences are different. Previous research found that perceived injunctive norm moderated the relationship between perceived descriptive norm and behavior such that this relationship was stronger at a high level of perceived injunctive norm compared to a low level (Lee et al., 2007; Göckeritz et al., 2010; Pandon et al., 2016). This suggests that individuals do not necessarily follow perceived descriptive norm because this type of norm only suggests that many people perform the given action. Instead, only when they perceive most people approve the behavior (i.e., perceived injunctive norm), they likely comply with the perceived descriptive norm.

In addition, TNSB contends that the strength of normative influence is moderated by group proximity (Rimal and Lapinski, 2015). Individuals are more likely to comply with norms of the group to which they are psychologically close (Rimal and Lapinski, 2015), which empirical research has supported (Neighbors et al., 2010, 2013; Woolf et al., 2014; Yang and Zhao, 2017). For instance, Neighbors et al. (2010) found that college students’ identification with specific groups moderated the relationship between perceived drinking norm in that group and drinking behavior such that group identification made the influence of the perceived drinking norm in the target group stronger. Similarly, identification with other marijuana users enhanced the influence of perceived norm on marijuana use (Neighbors et al., 2013). Therefore, the perceived workout norm in one’s network should exhibit a stronger impact on workout behavior than the same norm in general society. Hence, the current research focuses on *perceived descriptive and injunctive workout norms in one’s social network*.

#### Perceived Workout Norm Cultivated by Social Network Sites

Previous research has extended TNSB by explaining what factors may shape perceived social norms, which then translate into engagement in the given behavior. One important predictor that affects perceived social norm is perceived exposure to media information about the given act (Litt and Stock, 2011; Zhang and Jemmott, 2014; Nesi et al., 2017; Rui and Stefanone, 2017; Yang and Zhao, 2017; Duong and Liu, 2019). Lapinski and Rimal (2005) argued that the cultivation theory could explain these findings. Cultivation theory maintains that media influence individuals by shaping their perception of the world, and heavy media users tend to perceive the world more closely to what media present (Gerbner, 1998). For example, as violence is prevalent on American television, heavy viewers were found to perceive the world more dangerous compared to light viewers (Ogles and Hoffner, 1987; Riddle et al., 2011).

Similarly, if individuals are exposed to a large amount of SNS posts on a given health behavior, these cues may be interpreted as signs that this behavior is prevalent among their social contacts. Indeed, Litt and Stock (2011) manipulated perceived descriptive norm of drinking by exposing participants to Facebook profiles, which feature alcohol consumption. Likewise, Duong and Liu (2019) manipulated perceived descriptive norm of e-cigarette by exposing participants to relevant news. Survey research also provided similar results. For instance, Zhang and Jemmott (2014) found that the unintentional exposure to sexual content online enhanced Chinese college students’ perceptions of descriptive and injunctive norms of sexual behavior. Yang and Zhao (2017) revealed that the exposure to pro-drinking information *via* SNSs predicted perceived injunctive drinking norm positively.

Notably, studies reviewed above tested exposure through self-report measures of the frequency of being exposed to the given information (Zhang and Jemmott, 2014; Yang and Zhao, 2017). This essentially measures perceived exposure rather than actual exposure. However, as reviewed earlier, individuals tend to act based on their interpretation of the reality (Perkins and Wechsler, 1996), so perceived rather than actual social norm exhibits a stronger effect on behavior. This perception of social norm is more likely shaped by perceived exposure to the relevant information. For example, if Alex thinks he is exposed to 50 drinking posts *via* Facebook every day but in reality he is exposed to only 20, he likely overestimates the prevalence of and the approval for drinking in his Facebook network. Thus, we use *perceived* exposure in this study. Based on the literature reviewed above, we propose,

*H1: Perceived exposure to workout-related SNS posts predicts perceived descriptive norm of workout in one’s network positively.*

In addition, although research provided a wealth of evidence on the effect of perceived exposure on perceived *injunctive* norms (Zhang and Jemmott, 2014; Nesi et al., 2017; Rui and Stefanone, 2017; Yang and Zhao, 2017), this direct relationship may not hold in the present study. As explained earlier, research reviewed above focuses on questionable behaviors. However, the extent to which a given behavior is deemed questionable depends on the situation (Cialdini et al., 1990). Marijuana use is inappropriate in the general society but can be deemed acceptable by a group of rebellious teenagers. Thus, SNS posts about these questionable behaviors provide signals about how members of one’s social network view the given behaviors. However, the pro-health nature of workout suggests that this behavior may be deemed appropriate in most situations, and this evaluation may not be related to the amount of information that expresses support to workout in one’s network. Thus, perceived exposure to SNS cues may not have a significant effect on perceived *injunctive* norm of workout.

The theory of normative social behavior posits that the perceived injunctive norm may mediate the relationship between perceived descriptive norm and behavior because individuals may view the prevalence cues as the signal that many individuals approve the given behavior (Rimal and Lapinski, 2015). In other words, if many people engage in certain behavior, they likely endorse that behavior. Hence, individuals perceiving to be exposed to a wealth of workout posts *via* SNSs may develop heightened levels of perceived descriptive norm of workout, which then translate into enhanced levels of perceived injunctive norm.

*H2: Perceived descriptive norm of workout in one’s network mediates the relationship between perceived exposure to workout-related SNS posts and perceived injunctive norm of workout in one’s network.*

Additionally, TNSB posits that both perceived descriptive and injunctive norms exhibit a main effect on the given behavior. This argument received empirical support in the context of workout (Okun et al., 2002; Lee, 2011). Thus,

*H3: Perceived (a) descriptive and (b) injunctive norms of workout in one’s network predict workout intention positively*.

### From Perceived Norm to Intention: The Role of Self-Efficacy

The theory of normative social behavior posited that perceived social norm did not always exhibit a linear relationship with behavior (Rimal and Lapinski, 2015). Instead, research on TNSB has identified a range of factors that can moderate the effect of perceived social norm on behavior (Dieterich et al., 2013; Yang and Zhao, 2017) such as self-efficacy (see Rimal and Lapinski, 2015 for a review).

As an important concept in social cognitive theory, self-efficacy remains a critical predictor of a wide range of human behaviors (see Strecher et al., 1986 for a review). Bandura (1977) conceptualized self-efficacy as one’s confidence in their ability to perform certain behavior. Individuals exhibiting high self-efficacy tend to set higher goals, demonstrate more commitment to goal accomplishment, perceive fewer behavioral barriers, and expect better outcomes (Bandura, 2004). When it comes to workout, empirical work shows that self-efficacy in workout plays a critical role in motivating individuals to engage in physical activities (Marcus et al., 1992; Oman and King, 1998; McAuley et al., 2006; Shin et al., 2006; Haas, 2011). For example, Marcus et al. (1992) found that employees’ self-efficacy in workout varied depending on their stage of preparedness for physical activity, which suggests that self-efficacy was positively associated with one’s readiness to exercise. Shin et al. (2006) found that self-efficacy in workout exhibited a strong influence on commitment to exercises among patients with osteoporosis and osteoarthritis.

In addition to the main effect, research revealed that self-efficacy could moderate the effect of perceived *descriptive* norm on behavior. Jang et al. (2013) found that self-efficacy in resisting alcohol consumption moderated the effect of perceived descriptive drinking norm on both drinking intention and behavior such that the normative influences were stronger among those reporting a low level of refusal self-efficacy. Zhao et al. (2018) revealed that self-efficacy in HIV testing moderated the effect of perceived social norm on HIV testing in the past 3 months and in lifetime such that self-efficacy enhanced the normative influence. Although Zhao et al. (2018) did not specify the type of perceived social norm in their study, their measure of this variable is consistent with the conceptualization of perceived descriptive norm. Therefore, similar to research suggesting that perceived injunctive norm enhanced the effect of perceived descriptive norm on the target behavior (Lee et al., 2007; Göckeritz et al., 2010; Pandon et al., 2016), these studies also suggest that individuals may not follow perceived descriptive norms blindly. Instead, individuals are more likely to follow perceived descriptive norms when they are confident in completing the given action.

*H4: Self-efficacy in workout moderates the effect of perceived descriptive norm of workout in one’s social network on workout intention such that this effect is stronger at a high level of self-efficacy compared to low*.

Furthermore, as mentioned earlier, we used two different measures of workout intention. CLT posits that the decision-making is influenced by one’s psychological distance of the target, which is positively related to the abstractness of the target (Trope and Liberman, 2010). The general measure of workout intention simply asks participants about their likelihood to engage in physical activities in the next month, without priming them to think about any details. In contrast, following Kim and Sundar (2012), the specific measure of workout intention asks participants about their likelihood to purchase coupons related to physical activities. This measure could prime participants to think about more detailed investments they have to make to physical activities, specifically money and time, which are identified as key barriers to physical activities (Spinney and Millward, 2010). Thinking about details related to the given behavior corresponds to the conceptualization of generality versus specificity that CLT conceptualized (Trope and Liberman, 2010). Taken together, the general measure suggests a higher level of psychological distance of workout intention compared to the specific measure.

Additionally, research on CLT shows that when individuals report a low level of psychological distance, their behavior is more strongly affected by their feasibility perception because the specificity of the target makes individuals focus more on detailed information (Liberman and Trope, 1998; Eyal et al., 2009; Chu and Yang, 2020). By contrast, when individuals report a high level of psychological distance, their behavior is more strongly affected by their desirability perception because the distance makes individuals unable to analyze details of the target, only developing an abstract mental construal (Liberman and Trope, 1998; Eyal et al., 2009; Chu and Yang, 2020). For example, Chu and Yang (2020) manipulated the psychological distance of climate change in their experiment. Their findings reveal that messages highlighting risks of the climate change motivated engagement in climate change mitigation actions more strongly at a high level of psychological distance (Chu and Yang, 2020). Conversely, when participants were primed to perceive climate change psychologically close, their engagement in climate change mitigation actions were more strongly motivated by messages highlighting efficacy of these actions (Chu and Yang, 2020).

Thus, when individuals are primed to evaluate their workout intention more specifically, they may give more considerations to whether they can complete the given task. This evaluation of their ability can be indicated by self-efficacy in workout. Conversely, when they are primed to assess their workout intention generally, they may give more considerations to the extent to which they think workout is desirable, which perceived injunctive norm may indicate. Hence,

*H5: The effect of self-efficacy on workout intention should be stronger for the specific measure compared to the general measure*.

*H6: The effect of perceived network injunctive norm on workout intention should be stronger for the general measure compared to the specific measure*.

## Materials and Methods

### Sample

We conducted an online survey and collaborated with a large company that provides sampling services in China. Participants had to be at least 18 years old and use at least one SNS platform at the time of data collection. A convenience sample of 840 responses was recruited. We excluded 33 incomplete responses, so the final sample size was 807. There were more female participants in the final sample (443, or 54.9%). A large portion of the participants finished 4-year college education (72.7%), followed by associate degree (13.9%), master’s or Ph.D. degree (8.4%), high school degree (4.1%), middle school (0.7%) and elementary school (0.1%). Almost 40% of the participants (39.7%) reported their monthly household income of 12,501–38,500 RMB (1890.82–5823.27 USD), followed by 8,001–12,500 RMB (1210.18–1890.67 USD, 26.4%), 5,001–8,000 RMB (756.42–1210.03 USD, 15.9%), 3,501–5,000 RMB (527.95–756.27 USD, 8.8%), 38,501–83,500 RMB (5823.43–12629.70 USD, 5.6%), 3,500 RMB or below (527.8 USD, 2.5%), and 83,501 RMB or above (12629.85 USD, 1.2%).

### Measures

*Perceived descriptive workout norm in one’s network* was measured by adapting the 3-item Likert scale assessing perceived descriptive norm of talking about organ donation with family (Park and Smith, 2007). Items include “exercise has become a lifestyle of most people that I know,” “most people that I know often exercise,” and “most people that I know exercise when they have time” (Cronbach’s α = 0.79, *M* = 3.65, SD = 0.83). *Perceived injunctive workout norm in one’s network* was also measured through Park and Smith (2007), which, however, exhibited a relatively low level of reliability (Cronbach’s α = 0.67, *M* = 4.24, SD = 0.58). Items include “most people that I know think it’s important to exercise/acknowledge the importance of exercise/think they should exercise when they have time.” *Self-efficacy in workout* was assessed through using the 5-item Likert scale on self-efficacy in exercises by Marcus et al. (1992). Items include “I’m confident I can participate in regular exercise when I’m tired/in a bad mood/I don’t have the time/on vacation/the weather is not good” (Cronbach’s α = 0.80, *M* = 3.53, SD = 0.76). All these variables were measured on a 5-point Likert scale (1 = strongly disagree, 5 = strongly agree).

Furthermore, *perceived exposure to workout-related SNS posts* was measured by using a 5-item scale. Participants were asked to assess how often they saw their family and friends shared photos and text-based statuses about their workout experiences, data about their workout, photos showing the accomplishments of their workout, and inspirational posts related to workout on a 5-point Likert scale (1 = never, 2 = seldom, 3 = sometimes, 4 = often, and 5 = always). Although this was a self-created scale, it received good results of reliability tests (Cronbach’s α = 0.77, *M* = 3.19, SD = 0.69).

We measured *workout intention* by using two different methods. First, we asked participants how likely they might engage in physical activities in the next month on a 5-point Likert question (1 = very unlikely, 5 = very likely; *M* = 4.36, SD = 0.76). In addition, Kim and Sundar (2012) measured the intention to engage in healthy behavior by asking participants to select coupons for health-facilitating and health-inhibiting products and services. Following their measurement, we assessed *workout intention* by asking participants to indicate their likelihood on a 5-point Likert scale (1 = very unlikely, 5 = very likely) to choose a coupon for gym membership (*M* = 3.83, SD = 0.99) and online training classes *via Keep*, a workout mobile app popular in China (*M* = 4.02, SD = 1.02). In order to minimize the influence of monetary costs, both coupons were indicated as 20% off. Table 1 presents descriptive analyses of and bivariate correlations between these variables. As measures of some variables were derived from established scales in English, the first author translated them into Chinese and the second author checked the validity.

**TABLE 1:** Descriptive statistics and zero-order correlations; means (SDs) presented along the diagonal.

	Perceived SNS exposure	Descriptive norm	Injunctive norm	Self-efficacy	Intention, exercise	Intention, gym	Intention, class
Perceived SNS exposure	3.19 (0.69)	0.49***	0.25***	0.35***	0.30***	0.23***	0.28***
Descriptive norm		3.65 (0.83)	0.43***	0.39***	0.32***	0.24***	0.28***
Injunctive norm			4.24 (0.58)	0.12***	0.22***	0.09*	0.12**
Self-efficacy				3.53 (0.76)	0.56***	0.27***	0.23***
Intention, exercise					4.36 (0.76)	0.30***	0.29***
Intention, gym						3.83 (0.99)	0.25***
Intention, class							4.02 (1.02)

***p < 0.001, **p < 0.01, *p < 0.05.

### Statistical Analysis

For all analyses, we controlled biological sex, age, education, and income. H1 and H2 proposed a simple mediation hypothesis. As Macro Process provides a convenient method to conduct mediation analyses, we tested them through Macro Process 3.4 developed by Andrew F. Hayes in Columbus (Hayes, 2017) with 5,000 bootstrapped samples.

**FIGURE 1:**
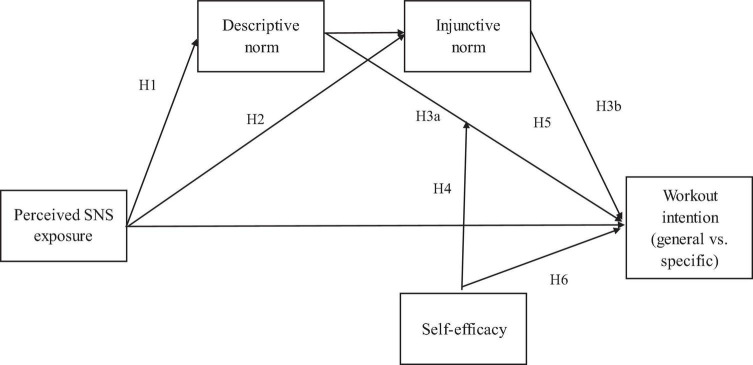
The hypothesized model.

Furthermore, as we proposed a moderated serial mediation model (Figure 1), H3 and H4 were examined within this model through the Lavaan package 6.8 *via* R. Simple effect tests were performed when a significant moderation effect emerged. The relationship between perceived descriptive norm and workout intention was compared between a high (one SD above the standardized mean) and low (one SD below the standardized mean) level of self-efficacy.

Finally, H5 and H6 were tested through the following formula (Clogg et al., 1995). *Z*-scores were computed to compare the effect of self-efficacy on the general workout intention and that on two specific workout intentions. If the absolute value of the *Z-*score exceeded 1.96, there were significant differences between these effects.


$$Z=\frac{\beta \,1-\beta \,2}{\sqrt{S\,E\,\beta \,1^{2}+S\,E\,\beta \,2^{2}}}$$

## Results

### Simple Mediation Analysis (H1–H2)

Perceived exposure to workout posts on SNSs predicted perceived descriptive norm of workout in one’s network (β = 0.47, *p* < 0.001; *R*^2^ = 0.26, *F*(5,801) = 57.16, *p* < 0.001) but did not exhibit a significant relationship with perceived injunctive norm of workout in one’s network (β = 0.05, *p* = 0.16; *R*^2^ = 0.20, *F*(6,800) = 32.46, *p* < 0.001). Perceived descriptive norm predicted perceived injunctive norm positively (β = 0.39, *p* < 0.001). The mediation between perceived SNS exposure and perceived injunctive norm of workout through perceived descriptive norm was significant [effect size: 0.27, 95% CI: (0.21, 0.33)]. H2 was supported. Among control variables, age predicted perceived descriptive norm positively (β = 0.12, *p* < 0.001) and education predicted perceived injunctive norm positively (β = 0.07, *p* < 0.05).

### Perceived Social Network Site Exposure, Perceived Workout Norm, Self-Efficacy, and General Workout Intention (H3–H4)

**FIGURE 2:**
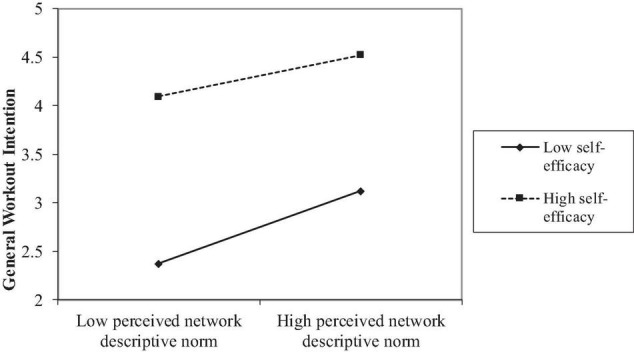
Self-efficacy moderated the relationship between perceived descriptive norm of workout and workout intention next month.

Perceived descriptive norm, self-efficacy, their interaction term, along with perceived injunctive norm, perceived SNS exposure and control variables explained 58.9% of total variances in the general workout intention. Perceived network descriptive (β = 0.29, *p* < 0.001, Table 2) and injunctive norms (β = 0.15, *p* < 0.001) predicted general workout intention positively, supporting H3a and H3b. In addition to a significant main effect (β = 0.78, *p* < 0.001), self-efficacy moderated the relationship between perceived network descriptive norm and general workout intention (β = − 0.08, *p* < 0.001) such that this relationship was significant at a low level of self-efficacy (β = 0.07, *p* < 0.041) but non-significant at a high level (*p* = 0.22, Figure 2). The result of the simple effect test was different from our prediction, so H4 was only partially supported.

**TABLE 2:** The two-step mediation model between perceived social network site (SNS) exposure and workout intention next month *via* perceived descriptive norm and perceived injunctive norm moderated by self-efficacy.

	Perceived descriptive norm of workout	Perceived injunctive norm of workout	Workout intention next month
Perceived SNS exposure	0.56***	0.04	0.09*
Sex	0.04	0.05	0.01
Education	0.03	0.06*	0.05
Income	0.04	0.01	0.06**
Age	0.015***	–0.001	0.002
Perceived descriptive norm of workout	NA	0.27***	0.29***
Perceived injunctive norm of workout	NA	NA	0.15***
Self-efficacy	NA	NA	0.78***
Descriptive × self-efficacy	NA	NA	−0.08***

***p < 0.001, **p < 0.01, *p < 0.05.

Among other variables, income (β = 0.06, *p* < 0.002) and perceived exposure (β = 0.09, *p* < 0.026) predicted the general workout intention. Perceived SNS exposure was positively related to perceived network descriptive norm (β = 0.47, *p* < 0.001) but not to perceived network injunctive norm (*p* = 0.15). Perceived network descriptive norm was positively associated with perceived network injunctive norm (β = 0.39, *p* < 0.001). These findings replicate the simple mediation analysis, thereby lending additional support to H1 and H2.

### Perceived Social Network Site Exposure, Perceived Workout Norm, Self-Efficacy, and Specific Workout Intention (H3–H4)

#### The Intention to Purchase the Gym Membership Coupon

**FIGURE 3:**
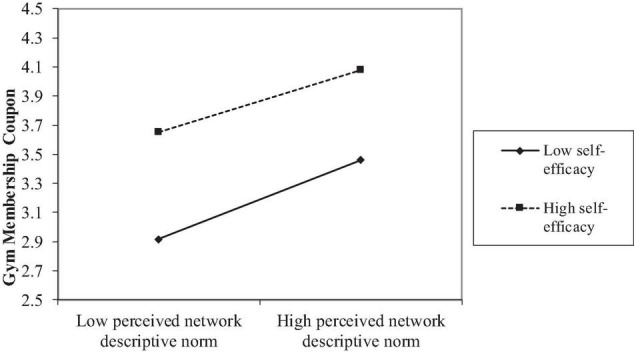
Self-efficacy moderated the relationship between perceived descriptive norm of workout and the intention to purchase the coupon for gym membership.

Perceived descriptive norm, self-efficacy, their interaction term, along with perceived injunctive norm, perceived SNS exposure and control variables explained 13.3% of total variances in the intention to purchase the gym membership coupon. While perceived network injunctive norm was not significantly related to the intention to purchase the gym membership coupon (*p* = 0.57), perceived network descriptive norm (β = 0.24, *p* < 0.001, Table 3) and self-efficacy (β = 0.34, *p* < 0.001) both exhibited a significant relationship with this intention. The moderation effect was significant as well (β = − 0.03, *p* < 0.001). The relationship between perceived network injunctive norm and the intention to purchase the gym membership coupon was stronger at a low level of self-efficacy (β = 0.16, *p* < 0.004) than a high level (β = 0.12, *p* < 0.05, Figure 3). Thus, H3a was supported, H3b was rejected, and H4 received partial support.

**TABLE 3:** The two-step mediation model between perceived social network site (SNS) exposure and the intention to purchase the gym membership coupon *via* perceived descriptive norm and perceived injunctive norm moderated by self-efficacy.

	Perceived descriptive norm of workout	Perceived injunctive norm of workout	Gym membership
Perceived SNS exposure	0.56***	0.04	0.155*
Sex	0.04	0.05	0.11
Education	0.03	0.06*	–0.05
Income	0.04	0.01	0.05
Age	0.015***	–0.001	0.001
Perceived descriptive norm of workout	NA	0.27***	0.24***
Perceived injunctive norm of workout	NA	NA	–0.04
Self-efficacy	NA	NA	0.34***
Descriptive × self-efficacy	NA	NA	−0.03***

***p < 0.001, *p < 0.05.

#### The Intention to Purchase the Online Training Class Coupon

**FIGURE 4:**
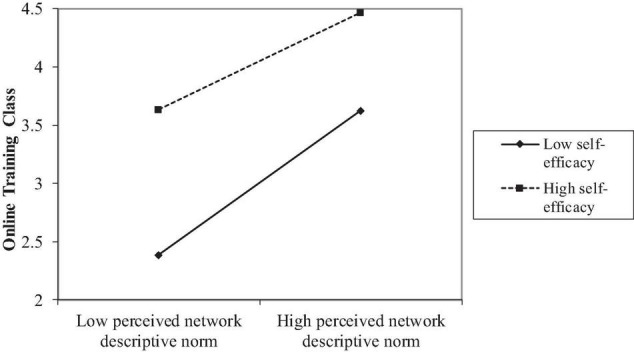
Self-efficacy moderated the relationship between perceived descriptive norm of workout and the intention to purchase the coupon for online training classes.

The proposed model explained 36.3% of total variances in the intention to choose the online training class coupon. Again, a non-significant relationship between perceived network injunctive norm and this intention was found (*p* = 0.66). Perceived network descriptive norm (β = 0.52, *p* < 0.001), self-efficacy (β = 0.52, *p* < 0.001), and their interaction term (β = − 0.10, *p* < 0.001, Table 4) all exhibited a significant relationship with the intention to purchase the online training class coupon. Specifically, the relationship between perceived network descriptive norm and this intention was significant at a low level of self-efficacy (β = 0.23, *p* < 0.001) but non-significant at a high level (*p* = 0.19, Figure 4). Again, H3a was supported, H3b was rejected, and H4 received partial support. Taken together, across both general and specific measures of the workout intention, H3a received full support but H4 was partially supported. H3b was supported for the general intention but rejected for the specific intention.

**TABLE 4:** The two-step mediation model between perceived social network site (SNS) exposure and the intention to purchase online training class *via* perceived descriptive norm and perceived injunctive norm moderated by self-efficacy.

	Perceived descriptive norm of workout	Perceived injunctive norm of workout	Online training class
Perceived SNS exposure	0.56***	0.04	0.25***
Sex	0.04	0.05	0.20**
Education	0.03	0.06*	0.08
Income	0.035	0.01	0.07*
Age	0.015***	–0.001	–0.00
Perceived descriptive norm of workout	NA	0.27***	0.52***
Perceived injunctive norm of workout	NA	NA	–0.03
Self-efficacy	NA	NA	0.52***
Descriptive × self-efficacy	NA	NA	−0.10***

***p < 0.001, **p < 0.01, *p < 0.05.

### Comparisons of the Effect of Self-Efficacy Between the General and Specific Intentions (H5–H6)

Following the formula mentioned above (Clogg et al., 1995), the effect of self-efficacy on the general workout intention was significantly larger than the intention to purchase the gym membership coupon (*Z* = 9.01) and the intention to purchase the online training class coupon (*Z* = 4.99). Although significant differences were found, the direction was opposite to what was proposed in H5. Thus, H5 was rejected.

Moreover, the effect of and perceived network injunctive norm on the general workout intention was significantly larger than the intention to purchase the gym membership coupon (*Z* = 2.63) and the intention to purchase the online training class coupon (*Z* = 2.38, Table 5). Hence, H6 was supported.

**TABLE 5:** Comparisons of the effect of self-efficacy and perceived injunctive norm on general versus specific workout intentions; standardized coefficients presented with SEs in the bracket.

	Self-efficacy	Differences, self-efficacy	Perceived injunctive norm	Differences, perceived injunctive norm
1. General intention	0.78 (0.028)	1–2: *Z* = 9.01 1–3: *Z* = 4.99	0.15 (0.04)	1–2: *Z* = 2.63 1–3: *Z* = 2.38
2. Coupon, gym membership	0.34 (0.04)		−0.04 (0.06)	
3. Coupon, online training class	0.52 (0.044)		−0.03 (0.064)	

## Discussion

As sharing details of daily life on SNSs such as workout experiences becomes increasingly prevalent, how workout-related posts may affect engagement of individuals in physical activities has attracted scholarly attention (Hamari and Koivisto, 2015; Vaterlaus et al., 2015; Burke and Rains, 2018; Raggatt et al., 2018; Peng et al., 2019). Drawing upon TNSB (Lapinski and Rimal, 2005; Rimal and Lapinski, 2015), the present investigation aims to explain the mechanism by which perceived exposure to workout-related SNS posts may affect physical activities through the lens of perceived social norm. Our findings demonstrate that perceived descriptive norm of workout in one’s network mediated the relationship between perceived exposure and perceived injunctive norm. In addition, self-efficacy in workout consistently moderated the effect of perceived descriptive norms on workout intention such that this effect was stronger at a low level of self-efficacy. Furthermore, the effects of self-efficacy and perceived injunctive norm was consistently larger on the general measure of the workout intention than the specific measure. These findings extend the scholarship on TNSB by explicating the relationships between perceived descriptive and injunctive norms and shed light on the relative strength of the motivating factors of workout in different situations.

### Major Findings and Implications

#### Social Network Site Exposure and Perceived Workout Norm

First, our findings show that the perceived exposure to workout-related SNS posts shaped perceived descriptive norm of workout in social network of an individual directly. In addition, after perceived descriptive norm was controlled, perceived SNS exposure did not exhibit a direct relationship with perceived injunctive norm. Instead, perceived descriptive norm mediated the relationship between perceived SNS exposure and perceived injunctive norm. This finding supports prior research that found positive relationships between perceived exposure and perceived descriptive norms of health behaviors (Litt and Stock, 2011; Zhang and Jemmott, 2014; Duong and Liu, 2019).

It is important to note that previous research found a significant relationship between perceived exposure and perceived injunctive norm of the given behavior (Zhang and Jemmott, 2014; Nesi et al., 2017; Rui and Stefanone, 2017; Yang and Zhao, 2017), which the present study did not replicate. One possible explanation is that those studies focus on questionable behaviors such as alcohol consumption (Nesi et al., 2017; Rui and Stefanone, 2017; Yang and Zhao, 2017) and sexual encounters (Zhang and Jemmott, 2014). Whether these behaviors are deemed proper is contextual (Cialdini et al., 1990). Thus, individuals may need SNS cues to evaluate its appropriateness in their social network. By contrast, for pro-health behaviors such as workout, this justification may not be needed, as in most situations workout is considered positive, regardless of the level of perceived exposure. This hence explains why the relationship between perceived SNS exposure and perceived injunctive norm of workout was insignificant. Instead, as Rimal and Lapinski (2015) posited, which also received empirical support by the current study, perceived injunctive norm may be a result of perceived descriptive norm. Therefore, we speculate that perceived descriptive norm of questionable behaviors may partially mediate the effect of perceived exposure on perceived injunctive norm, whereas this might be a full mediation for pro-health behaviors. Future research can test this speculation.

Due to the cross-sectional design of the present study, we cannot make causal argument that perceived descriptive norm causes perceived injunctive norm. However, we argue that the opposite direction may not hold theoretically. Although many people may endorse certain behavior (i.e., perceived injunctive norm), they may not necessarily do so (i.e., perceived descriptive norm) because people may lack the skills, resources, or motivation to perform the given act despite their approval of the behavior (Yzer, 2012). This especially applies to workout, which requires physical and time investments. Hence, the mediation between perceived SNS exposure and perceived injunctive norm *via* perceived descriptive norm is more theoretically valid.

In addition, one limitation regarding the effect of perceived SNS exposure on perceived workout norm is that individuals’ perception of workout norm may come from multiple sources. Apart from SNSs, interpersonal discussions, direct observations, and traditional mass media exposure may all shape their perceived workout norm. In fact, when other channels are considered, SNS exposure may not always shape perceived descriptive norm of the given health behavior (Rui and Stefanone, 2017; Yang and Zhao, 2017). Hence, we cannot make arguments about the *exclusive* impact of SNS exposure on perceived workout norm, and cautions are required to interpret our findings.

#### Perceived Workout Norm, Self-Efficacy, and Intention

The present investigation also sheds light on how perceived workout norm—both descriptive and injunctive—may affect workout intention and how self-efficacy might be positioned as a moderator in the relationship between perceived descriptive norm and workout intention. Several interesting patterns have emerged from our findings which are relatively consistent across all three measures of workout intention.

First, perceived *descriptive* norm enhanced workout intention, regardless of the measure. One possible explanation is that descriptive norm is associated with prevalence (Cialdini et al., 1990; Park and Smith, 2007). Therefore, individuals driven by perceived descriptive norm to engage in certain behaviors may expect to fulfill social needs while performing these acts. Workout is such an example (Snyder, 1970). Going to the gym can satisfy their need for socialization. As for taking online training classes, we specified that the classes were purchased through *Keep*, a workout mobile app popular in China. Users can share their exercise experiences with others through this app, so this option for physical activities can also gratify social needs. This finding hence corresponds to the wealth of empirical evidence, which demonstrates the peer influence on workout (Lee, 2011; Yun and Silk, 2011).

In contrast, perceived *injunctive* norm predicted the likelihood to engage in physical activities in the next month rather than the likelihood to purchase the coupons for gym membership and online training classes. As explained earlier, CLT posits that individuals’ decisions are more driven by desirability perception when they exhibit a high level of psychological distance, whereas their decisions are more driven by feasibility perception when their psychological distance is low (Liberman and Trope, 1998; Eyal et al., 2009; Chu and Yang, 2020). Moreover, psychological distance is associated with the level of abstractness of one’s mental representation of the target (Trope and Liberman, 2010). Hence, at the general level, one’s workout decisions may be driven by whether the given act is appropriate, indicated by perceived injunctive norm. However, when individuals are primed to consider detailed commitments that they have to make to workout, feasibility becomes the key predictor that affects their decisions. For example, participants may not think it is worthwhile to purchase these coupons given alternative free options for physical activities. They might also have to evaluate if they can make time commitment to these paid choices of physical activities. Hence, perceived injunctive norm of workout in their network did not affect their decisions to purchase these coupons.

Later, self-efficacy in workout consistently predicted all measures of workout intention. This suggests that compared to perceived norms, self-efficacy might be more important in encouraging workout. After all, workout requires skills, time, and resources. Thus, when it comes to evaluating the likelihood to engage in physical activities, individuals may give more considerations to whether they have the required ability and resources, rather than simply follow what most other people do.

Additionally, self-efficacy moderated the effect of perceived descriptive norm on all three workout intentions, but it dampened the norm influence, opposite to our prediction. This suggests that perceived descriptive norm and self-efficacy may represent two routes to motivating workout, which are not complementary. Individuals may follow the crowd to engage in workout, and this peer influence may not be driven by self-efficacy. Alternatively, they may be intrinsically motivated to exercise because of their confidence, which has nothing to do with peer influence.

#### General Versus Specific Workout Intention: Self-Efficacy and Perceived Injunctive Norm

Finally, we compared the effects of self-efficacy and perceived injunctive norm on the specific versus general measure of workout intention. As explained earlier, our findings show that the perceived injunctive norm predicted the general workout intention but not the specific, which supports CLT research demonstrating a stronger effect of desirability perception on behavior when individuals are primed to think generally about it (Liberman and Trope, 1998; Eyal et al., 2009; Chu and Yang, 2020).

However, opposite to the hypothesis, self-efficacy exhibited a stronger effect on the general workout intention than the specific. One possible explanation is that other variables may influence participants’ decisions of coupon purchases such as money. Indeed, money is identified as a key barrier to physical activities (Spinney and Millward, 2010). However, we controlled income as a way to consider money influence on the specific workout intention, and the *post hoc* analysis shows that the effects of income did not differ significantly between the general and specific measures of workout intention^1^.

We speculate that the current measure of self-efficacy in workout may not be a good indicator of feasibility perception of workout. This measure shows one’s self-rated ability to tackle obstacles to workout including a tight schedule, exhaustion, laziness, and bad mood (Marcus et al., 1992, see the Appendix). However, concerns over money and physical strength may also be involved in the evaluation of feasibility of workout.

Regardless, to the best of our knowledge, these findings are among the limited research that compared the relative strengths of the predictors of workout intention in a general versus specific situation. Although our hypotheses did not receive full support, the result of perceived injunctive norm suggests that the perception of most others’ approval can be an important factor that motivates individuals’ decisions of workout. However, this perception may not be priority when individuals start to consider details about their preparations for workout.

This finding along with results mentioned provides important practical implications for workout campaigns. Practitioners can collaborate with workout apps and encourage individuals to share their workout experiences on SNSs. This could directly elevate individuals’ perceived descriptive norms of workout in their network and indirectly enhance their perceived injunctive norms. However, this social norm approach may only be effective for those who have not had sufficient considerations of exercises. For those who have thought about details of physical activities or started to engage in physical activities, campaigns should focus on addressing their obstacles to workout and elevate their self-efficacy.

### Limitations and Future Directions

Several limitations of the present study are worth mentioning. A major problem of this study is its cross-sectional design. Therefore, when it comes to the effect of SNS exposure on perceived workout norm, we cannot rule out potential influences of other sources, and more robust evidence is needed to confirm the mediation result we found. Experiment or longitudinal research is needed for future inquiries.

Second, the present study was conducted with a convenience sample of Chinese individuals. This could limit the internal and external validity of the current findings. Cross-cultural comparisons with random samples are needed.

Furthermore, as mentioned before, our measure of perceived injunctive workout norm did not demonstrate a good result of reliability. This might threaten the validity of our measure and increase the chance of type II error.

In addition, following Kim and Sundar (2012), we used a behavioral measure as a proxy for assessing individuals’ intention to engage in physical activities. This measure can prime participants to consider more details related to workout when evaluating their workout intention. Although additional factors such as monetary costs may be introduced, money is indeed a key barrier to physical activities (Spinney and Millward, 2010). However, the validity of this measure needs to be re-examined, and purchasing workout-related coupons may not be the only way to measure the specific intention of workout.

In addition to addressing these limitations, future research may benefit from following directions. In terms of understanding how norms develop, future research can examine the relationship between collective norm and perceived norm. As Miller and Prentice (1996) argued, one source of perceived norm is direct observation. Thus, individuals may develop perceived norm based on their observation of collective norm. Hence, perceived norm may be a result of collective norm. Additionally, research is needed to understand how collective norm forms. In addition to individual behaviors, institutional, national policies, or culture may contribute to the formation of collective norms.

Moreover, as explained earlier, our study suggests that whether the given behavior is pro-health may affect the relationship between SNS exposure and perceived injunctive norm, which future research should test. Furthermore, systematic investigations are needed to understand the attributes of behaviors and examine how these behavioral attributes may moderate normative influences.

## Conclusion

The present research investigated how SNSs may affect individuals’ intention to engage in physical activities from the perspective of perceived social norm. Our findings explicate the relationship and difference between perceived descriptive and injunctive norms and highlight the important role that self-efficacy plays in motivating workout. Additionally, perceived injunctive norm was a significant predictor of general workout intention but became non-significant when individuals were primed to consider details about workout. Therefore, social norm can be a valuable approach to campaigns on workout in which SNSs can play a critical role, but the relative strength of perceived norm may depend on individuals’ self-efficacy and the specificity of the context.

## Data Availability Statement

The raw data supporting the conclusions of this article will be made available by the authors, without undue reservation.

## Ethics Statement

The studies involving human participants were reviewed and approved by Research Ethics Committee at South China University of Technology. The patients/participants provided their written informed consent to participate in this study.

## Author Contributions

JR: responsible for conceptualization, questionnaire design, data analysis, and manuscript writing. SL: responsible for conceptualization, questionnaire design, data collection, and editing. Both authors contributed to the article and approved the submitted version.

## Conflict of Interest

The authors declare that the research was conducted in the absence of any commercial or financial relationships that could be construed as a potential conflict of interest.

## Publisher’s Note

All claims expressed in this article are solely those of the authors and do not necessarily represent those of their affiliated organizations, or those of the publisher, the editors and the reviewers. Any product that may be evaluated in this article, or claim that may be made by its manufacturer, is not guaranteed or endorsed by the publisher.

## Footnotes

1 We compared the effect of income on the general (β = 0.064, SE = 0.02) versus specific workout intention (gym membership: β = 0.05, SE = 0.03; online training class: β = 0.07, SE = 0.03). However, no significant differences were found between the general measure and the intention to purchase the gym membership coupon (*Z* = − 0.17) and between the general measure and the intention to purchase the coupon for online training classes (*Z* = − 0.47).

## References

[B1] Ajzen I. Fishbein M. (1980). *Understanding Attitudes and Predicting Social Behavior.* Hoboken, NJ: Prentice Hall.

[B2] Bandura A. (1977). Self-efficacy: toward a unifying theory of behavioral change. *Psychol. Rev.* 84 191–215. 10.1037/0033-295x.84.2.191 847061

[B3] Bandura A. (2004). Health promotion by social cognitive means. *Health Educ. Behav.* 31 143–164. 10.1177/1090198104263660 15090118

[B4] Borsari B. Carey K. B. (2001). Peer influences on college drinking: a review of the research. *J. Subst. Abuse* 13 391–424. 10.1016/s0899-3289(01)00098-0 11775073

[B5] Borsari B. Carey K. B. (2003). Descriptive and injunctive norms in college drinking: a meta-analytic integration. *J. Stud. Alcohol* 64 331–341. 10.15288/jsa.2003.64.331 12817821 PMC2431131

[B6] Burke T. J. Rains S. A. (2018). The paradoxical outcomes of observing others’ exercise behavior on social network sites: friends’ exercise posts, exercise attitudes, and weight concern. *Health Commun.* 34 475–483. 10.1080/10410236.2018.1428404 29364740

[B7] Chu H. Yang J. (2020). Risk or efficacy? How psychological distance influences climate change engagement. *Risk Anal.* 40 758–770. 10.1111/risa.13446 31957904

[B8] Cialdini R. B. Reno R. R. Kallgren C. A. (1990). A focus theory of normative conduct: recycling the concept of norms to reduce littering in public places. *J. Pers. Soc. Psychol.* 58 1015–1026. 10.1037/0022-3514.58.6.1015

[B9] Clogg C. C. Petkova E. Haritou A. (1995). Statistical methods for comparing regression coefficients between models. *Am. J. Sociol.* 100 1261–1293. 10.1086/230638

[B10] Dieterich S. E. Stanley L. R. Swaim R. C. Beauvais F. (2013). Outcome expectancies, descriptive norms, and alcohol use: American Indian and White adolescents. *J. Prim. Prevent.* 34 209–219. 10.1007/s10935-013-0311-6 23754535 PMC8061310

[B11] Duong H. T. Liu J. (2019). Vaping in the news: the influence of news exposure on perceived e-cigarette use norms. *Am. J. Health Educ.* 50 25–39. 10.1080/19325037.2018.1548315

[B12] Eyal T. Sagristano M. D. Trope Y. Liberman N. Chaiken S. (2009). When values matter: expressing values in behavioral intentions for the near vs. distant future. *J. Exp. Soc. Psychol.* 45 35–43. 10.1016/j.jesp.2008.07.023 21822329 PMC3150799

[B13] Gerbner G. (1998). Cultivation analysis: an overview. *Mass Commun. Soc.* 1 175–194. 10.1080/15205436.1998.9677855

[B14] Göckeritz S. Schultz P. W. Rendón T. Cialdini R. B. Goldstein N. J. Griskevicius V. (2010). Descriptive normative beliefs and conservation behavior: the moderating roles of personal involvement and injunctive normative beliefs. *Eur. J. Soc. Psychol.* 40 514–523. 10.1002/ejsp.643

[B15] Haas B. K. (2011). Fatigue, self-efficacy, physical activity, and quality of life in women with breast cancer. *Cancer Nurs.* 34 322–334. 10.1097/ncc.0b013e3181f9a300 21116178

[B16] Hamari J. Koivisto J. (2015). “Working out for likes”: an empirical study on social influence in exercise gamification. *Comput. Hum. Behav.* 50 333–347. 10.1016/j.chb.2015.04.018

[B17] Hayes A. (2017). *Introduction to Mediation, Moderation, and Conditional Process Analysis: A Regression-Based Approach (The Second Edition).* New York, NY: Guilford Press.

[B18] Jang S. A. Rimal R. N. Cho N. (2013). Normative influences and alcohol consumption: the role of drinking refusal self-efficacy. *Health Commun.* 28 443–451. 10.1080/10410236.2012.691455 22809467

[B19] Kemp S. (2020). *Digital 2020: July Global Statshot.* Available online at: https://datareportal.com/reports/digital-2020-july-global-statshot (accessed November 20, 2020).

[B20] Kim Y. Sundar S. S. (2012). Visualizing ideal self vs. actual self through avatars: impact on preventive health outcomes. *Comput. Hum. Behav.* 28 1356–1364. 10.1016/j.chb.2012.02.021

[B21] Lapinski M. K. Rimal R. V. (2005). An explication of social norms. *Commun. Theory* 15 127–147. 10.1111/j.1468-2885.2005.tb00329.x

[B22] Leary M. R. Baumeister R. F. (2000). The nature and function of self-esteem: sociometer theory. *Adv. Exp. Soc. Psychol.* 32 1–62. 10.1016/s0065-2601(00)80003-9

[B23] Lee C. M. Geisner I. M. Lewis M. A. Neighbors C. Larimer M. E. (2007). Social motives and the interaction between descriptive and injunctive norms in college student drinking. *J. Stud. Alcohol Drugs* 68 714–721. 10.15288/jsad.2007.68.714 17690805

[B24] Lee H. (2011). The role of descriptive norm within the theory of planned behavior in predicting Korean Americans’ exercise behavior. *Psychol. Rep.* 109 208–218. 10.2466/06.07.PR0.109.4.208-218 22049662

[B25] Liberman N. Trope Y. (1998). The role of feasibility and desirability considerations in near and distant future decisions: a test of temporal construal theory. *J. Pers. Soc. Psychol.* 75 5–18. 10.1037/0022-3514.75.1.5

[B26] Litt D. M. Stock M. L. (2011). Adolescent alcohol-related risk cognitions: the roles of social norms and social networking sites. *Psychol. Addict. Behav.* 25 708–713. 10.1037/a0024226 21644803

[B27] Liu Y. Kashian N. (2020). Sharing workout experiences on social networking sites: its moderating factors and well-being outcomes. *Health Commun.* 36 1309–1319. 10.1080/10410236.2020.1750774 32316777

[B28] Marcus B. H. Selby V. C. Niaura R. S. Rossi J. S. (1992). Self-efficacy and the stages of exercise behavior change. *Res. Q. Exerc. Sport* 63 60–66. 10.1080/02701367.1992.10607557 1574662

[B29] McAuley E. Konopack J. F. Morris K. S. Motl R. W. Hu L. Doerksen S. E. (2006). Physical activity and functional limitations in older women: influence of self-efficacy. *J. Gerontol. Ser. B* 61 270–277. 10.1093/geronb/61.5.P270 16960230

[B30] Miller D. T. Prentice D. A. (1996). “The construction of social norms and standards,” in *Social Psychology: Handbook of Basic Principles*, eds Higgins E. T. Kruglanski A. W. (New York, NY: Guilford Press), 799–829.

[B31] Mollen S. Rimal R. N. Ruiter R. A. Kok G. (2013). Healthy and unhealthy social norms and food selection: findings from a field-experiment. *Appetite* 65 83–89. 10.1016/j.appet.2013.01.020 23402712

[B32] Neighbors C. Foster D. W. Walker D. D. Kilmer J. R. Lee C. M. (2013). Social identity as a moderator of the association between perceived norms and marijuana use. *J. Stud. Alcohol Drugs* 74 479–483. 10.15288/jsad.2013.74.479 23490578 PMC3602363

[B33] Neighbors C. LaBrie J. W. Hummer J. F. Lewis M. A. Lee C. M. Desai S. (2010). Group identification as a moderator of the relationship between perceived social norms and alcohol consumption. *Psychol. Addict. Behav.* 24 522–528. 10.1037/a0019944 20853938 PMC2946396

[B34] Nesi J. Rothenberg W. A. Hussong A. M. Jackson K. M. (2017). Friends’ alcohol-related social networking site activity predicts escalations in adolescent drinking: mediation by peer norms. *J. Adolesc. Health* 60 641–647. 10.1016/j.jadohealth.2017.01.009 28325545 PMC6402495

[B35] Ogles R. M. Hoffner C. (1987). Film violence and perceptions of crime: the cultivation effect. *Ann. Int. Commun. Assoc.* 10 384–394. 10.1080/23808985.1987.11678653

[B36] Okun M. A. Karoly P. Lutz R. (2002). Clarifying the contribution of subjective norm to predicting leisure-time exercise. *Am. J. Health Behav.* 26 296–305. 10.5993/ajhb.26.4.6 12081362

[B37] Oman R. F. King A. C. (1998). Predicting the adoption and maintenance of exercise participation using self-efficacy and previous exercise participation rates. *Am. J. Health Promot.* 12 154–161. 10.4278/0890-1171-12.3.154 10176088

[B38] Pandon A. A. Rimal R. N. Jernigan D. Siegel M. Dejong W. (2016). Tapping into motivations for drinking among youth: normative beliefs about alcohol use among underage drinkers in the United States. *J. Health Commun.* 21 1079–1087. 10.1080/10810730.2016.1222030 27668832 PMC5155584

[B39] Park H. S. Smith S. W. (2007). Distinctiveness and influence of subjective norms, personal descriptive and injunctive norms, and societal descriptive and injunctive norms on behavioral intent: a case of two behaviors critical to organ donation. *Hum. Commun. Res.* 33 194–218. 10.1111/j.1468-2958.2007.00296.x

[B40] Peng C. Wu T. Chen Y. Atkin D. J. (2019). Comparing and modeling via social media: the social influences of fitspiration on male Instagram users’ work out intention. *Comput. Hum. Behav.* 99 156–167. 10.1016/j.chb.2019.05.011

[B41] Perkins H. Wechsler H. (1996). Variation in perceived college drinking norms and its impact on alcohol abuse: a nationwide study. *J. Drug Issues* 26 961–974. 10.1177/002204269602600413

[B42] Pinkerton S. Tobin J. L. Querfurth S. C. Pena I. M. Wilson K. S. (2017). “Those sweet, sweet likes”: sharing physical activity over social network sites. *Comput. Hum. Behav.* 69 128–135. 10.1016/j.chb.2016.12.028

[B43] Raggatt M. Wright C. J. C. Carrotte E. Jenkinson R. Mulgrew K. Prichard I. (2018). “I aspire to look and feel healthy like the posts convey”: engagement with fitness inspiration on social media and perceptions of its influence on health and wellbeing. *BMC Public Health* 18:1002. 10.1186/s12889-018-5930-7 30097034 PMC6086030

[B44] Riddle K. Potter W. J. Metzger M. J. Nabi R. L. Linz D. G. (2011). Beyond cultivation: exploring the effects of frequency, recency, and vivid autobiographical memories for violent media. *Media Psychol.* 14 168–191. 10.1080/15213269.2011.573464

[B45] Rimal R. N. Lapinski M. K. (2015). A re-explication of social norms, ten years later. *Commun. Theory* 25 393–409. 10.1111/comt.12080

[B46] Rimal R. N. Real K. (2003). Understanding the influence of perceived norms on behaviors. *Commun. Theory* 13 184–203. 10.1111/j.1468-2885.2003.tb00288.x

[B47] Rui J. R. Stefanone M. A. (2017). “Assessing drinking norms from attending drinking events and social network site use,” in *Proceedings of the 50th Hawaii International Conference on System Science*, New York, NY, 3736–3745.

[B48] Shin Y. H. Hur H. K. Pender N. J. Jang H. J. Kim M. S. (2006). Exercise self-efficacy, exercise benefits and barriers, and commitment to a plan for exercise among Korean women with osteoporosis and osteoarthritis. *Int. J. Nurs. Stud.* 43 3–10. 10.1016/j.ijnurstu.2004.10.008 16326159

[B49] Snyder E. E. (1970). Aspects of socialization in sports and physical education. *Quest* 14 1–7. 10.1080/00336297.1970.10519683

[B50] Spinney J. Millward H. (2010). Time and money: a new look at poverty and the barriers to physical activity in Canada. *Soc. Indic. Res.* 99 341–356. 10.1007/s11205-010-9585-8

[B51] Staunton M. Louis W. R. Smith J. R. Terry D. J. McDonald R. I. (2014). How negative descriptive norms for healthy eating undermine the effects of positive injunctive norms. *J. Appl. Soc. Psychol.* 44 319–330. 10.1111/jasp.12223

[B52] Strecher V. J. DeVellis B. M. Becker M. H. Rosenstock I. M. (1986). The role of self-efficacy in achieving health behavior change. *Health Educ. Q.* 13 73–91. 10.1177/109019818601300108 3957687

[B53] Trope Y. Liberman N. (2010). Construal-level theory of psychological distance. *Psychol. Rev.* 117 440–463. 10.1037/a0018963 20438233 PMC3152826

[B54] Vaterlaus J. M. Patten E. V. Roche C. Young J. A. (2015). #Gettinghealthy: the perceived influence of social media on young adult health behaviors. *Comput. Hum. Behav.* 45 151–157. 10.1016/j.chb.2014.12.013

[B55] Woolf J. Rimal R. N. Sripad P. (2014). Understanding the influence of proximal networks on high school athletes’ intentions to use androgenic anabolic steroids. *J. Sport Manag.* 28 8–20. 10.1123/jsm.2013-0046

[B56] Yang B. Zhao X. (2017). TV, social media, and college students’ binge drinking intentions: moderated mediation models. *J. Health Commun.* 23 61–71. 10.1080/10810730.2017.1411995 29265924

[B57] Yun D. Silk K. J. (2011). Social norms, self-identity, and attention to social comparison information in the context of exercise and healthy diet behavior. *Health Commun.* 26 275–285. 10.1080/10410236.2010.549814 21400325

[B58] Yzer M. (2012). “The integrative model of behavioral prediction as a tool for designing health messages,” in *Health Communication Message Design: Theory and Practice*, ed. Cho H. (Thousand Oaks, CA: Sage), 21–40.

[B59] Zhang J. Jemmott J. B. (2014). Unintentional exposure to online sexual content and sexual behavior intentions among college students in China. *Asia Pac. J. Public Health* 27 561–571. 10.1177/1010539514562446 25527202

[B60] Zhao P. Liu L. Zhang Y. Cheng H. Cao B. Liu C. (2018). The interaction between HIV testing social norms and self-efficacy on HIV testing among Chinese men who have sex with men: results from an online cross-sectional study. *BMC Infect. Dis.* 18:541. 10.1186/s12879-018-3454-5 30376818 PMC6208016

